# O Índice Nutricional Prognóstico está Associado ao Grau de Circulação Colateral Coronariana em Pacientes com Angina Estável e Oclusão Total Crônica

**DOI:** 10.36660/abc.20230765

**Published:** 2024-03-21

**Authors:** Kerim Esenboga, Alparslan Kurtul, Yakup Yunus Yamanturk, Volkan Kozluca, Eralp Tutar

**Affiliations:** 1 Ankara University Faculty of Medicine Ankara Turquia Ankara University Faculty of Medicine – Cardiology, Ankara – Turquia; 2 Mustafa Kemal University Tayfur Ata Sokmen Faculty of Medicine Department of Cardiology Hatay Turquia Mustafa Kemal University Tayfur Ata Sokmen Faculty of Medicine – Department of Cardiology, Hatay – Turquia

**Keywords:** Circulação Colateral, Avaliação Nutricional, Inflamação

## Abstract

**Fundamento:**

A circulação colateral coronária (CCC) pode efetivamente melhorar o suprimento sanguíneo miocárdico para a área de OCT (oclusão coronariana total crônica) e pode, assim, melhorar o prognóstico de pacientes com síndrome coronariana estável (SCE). O grau de inflamação e alguns marcadores de inflamação foram associados ao desenvolvimento de colaterais.

**Objetivo:**

Investigar se o índice nutricional prognóstico (INP) tem associação com o desenvolvimento de CCC em pacientes com SCE.

**Métodos:**

Um total de 400 pacientes com SCE com presença de OTC em pelo menos uma importante artéria coronária epicárdica foi incluído neste estudo. Os pacientes foram divididos em dois grupos de acordo com o escore Rentrop. Escores de 0 a 1 foram considerados CCC pouco desenvolvidas e escores de 2 a 3 foram aceitos como CCC bem desenvolvidas. A significância estatística foi definida como um valor p < 0,05 para todas as análises.

**Resultados:**

A média de idade da coorte do estudo foi de 63±10 anos; 273 (68,3%) eram do sexo masculino. O grupo CCC pouco desenvolvido apresentou um nível de INP significativamente mais baixo em comparação com o grupo CCC bem desenvolvido (38,29±5,58 vs 41,23±3,85, p<0,001). Na análise multivariada, o INP (odds ratio 0,870; intervalo de confiança de 95% 0,822-0,922; p<0,001) foi um preditor independente de CCC pouco desenvolvida.

**Conclusão:**

O INP pode ser utilizado como um dos preditores independentes da formação do CCC. Foi positivamente associado ao desenvolvimento de colaterais coronárias em pacientes com SCE com OTC.

**Figure:**
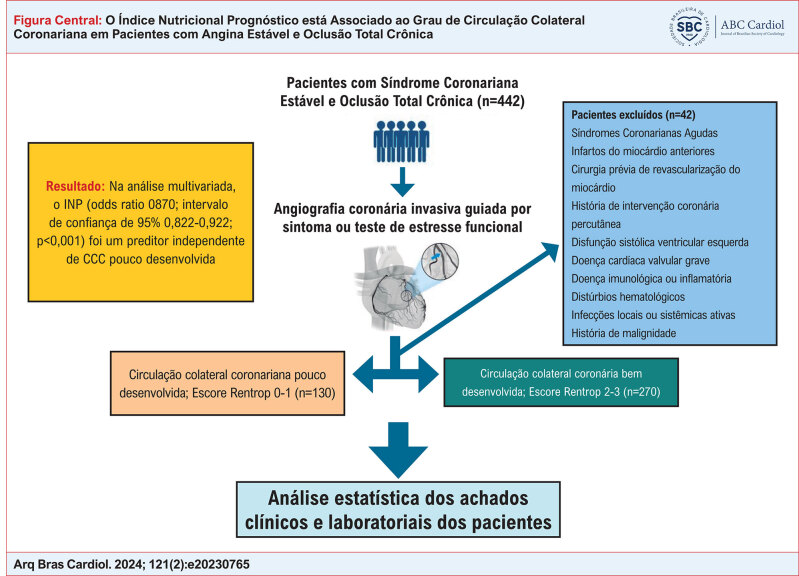
Resumo do desenho e resultados do estudo.

## Introdução

A circulação colateral coronária (CCC) geralmente é um mecanismo adaptativo durante a isquemia miocárdica crônica para fornecer fluxo sanguíneo no território isquêmico.^1^ O desenvolvimento da CCC pode manter o fluxo sanguíneo coronariano até certo ponto, aliviar queixas anginosas, proporcionar preservação miocárdica diante de situações agudas. isquemia, preservar a função miocárdica e melhorar a sobrevida em pacientes com síndrome coronariana estável (SCE).^2^ Embora os mecanismos exatos de desenvolvimento de colateralização coronariana em pacientes com SCE ainda sejam conflitantes e inconclusivos, vários estudos sugerem que marcadores inflamatórios sistêmicos, como a relação neutrófilos/linfócitos (NLR) e a proteína C reativa (PCR) estão associadas ao desenvolvimento de colaterais.^3 , 4^

Estudos anteriores relataram que os estados imunológico e nutricional estão intimamente associados ao desenvolvimento, progressão e prognóstico de doenças cardiovasculares.^5 , 6^ Recentemente, a investigação do índice nutricional prognóstico (INP) tornou-se muito popular. O INP, calculado a partir do nível sérico de albumina e da contagem total de linfócitos no sangue periférico, é um índice que indica inflamação crônica, estado do sistema imunológico e nutricional e tem valor prognóstico em vários tipos de câncer.^7 , 8^ Recentemente, muitos estudos relataram que o INP está intimamente relacionado com doenças cardiovasculares, e um INP mais baixo está significativamente associado ao aumento de resultados clínicos adversos, incluindo mortalidade em pacientes com SCE, insuficiência cardíaca, dissecção aórtica e infarto agudo do miocárdio.^9 – 14^ No entanto, a associação entre INP e CCC ainda não foi investigada.

O objetivo do presente estudo é, portanto, explorar se existe uma correlação entre o INP e o grau de desenvolvimento de CCC em pacientes com SCE.

## Métodos

### Pacientes do estudo

A população do estudo foi composta por 442 pacientes com SCE que foram submetidos à angiografia coronariana (AC) e foram detectados com oclusão coronariana total (OCT) crônica em qualquer artéria coronária no período de maio de 2017 a maio de 2021. A AC foi realizada para investigar doença arterial coronariana oclusiva com base em dados clínicos, sinais como presença de sintomas anginosos típicos, testes ergométricos anormais ou suspeitos e achados anormais de cintilografia de perfusão miocárdica sugestivos de isquemia miocárdica. Pacientes com infartos do miocárdio prévios, cirurgia de revascularização miocárdica prévia, história de intervenção coronária percutânea, insuficiência cardíaca significativa (fração de ejeção do ventrículo esquerdo [FEVE] ≤35%), doença renal crônica grave (taxa de filtração glomerular estimada [TFGe] <30 mL/min/1,73 m²), disfunção valvar grave, doença hepática crônica grave, distúrbios hematológicos, doença imunológica/infecciosa/inflamatória ativa e pacientes com malignidade não foram incluídos no estudo. Quarenta e dois pacientes foram excluídos devido aos critérios de exclusão (10 pacientes com história prévia de infarto do miocárdio ou intervenção coronária percutânea; 10 pacientes com doença renal crônica grave; 5 pacientes com insuficiência cardíaca; 5 pacientes com valvopatia; 3 pacientes com doenças inflamatórias; 2 pacientes com malignidade; 5 pacientes com doença hepática crônica; e 2 pacientes com distúrbios hematológicos). Os 400 pacientes restantes foram incluídos na análise final. No total, 130 pacientes foram alocados no grupo CCC ruim, enquanto 270 pacientes foram alocados no grupo CCC bom.

Dados clínicos basais e fatores de risco de doença cardiovascular aterosclerótica foram anotados para todos os pacientes. Foi considerada hipertensão arterial naqueles pacientes com medidas repetidas de pressão arterial >140/90 mmHg ou que já faziam uso de anti-hipertensivos. O diabetes mellitus foi descrito como tendo um nível de glicose sérica em jejum > 126 mg/dL e glicose pós-prandial > 200 mg/dL em medições repetidas ou uso atual de terapia antidiabética. Um nível elevado de colesterol total superior a 200 mg/dL e/ou uso de medicamentos anti-hiperlipidêmicos foram utilizados para caracterizar hipercolesterolemia. A definição de história familiar de doença arterial coronariana (DAC) foi história de DAC ou morte súbita cardíaca em parentes de primeiro grau com idade inferior a 55 anos para homens e 65 anos para mulheres.

Amostras de sangue venoso antecubital foram coletadas após pelo menos 12 horas de jejum antes da AC. O analisador Beckman Colter foi utilizado para medir os parâmetros rotineiros do hemograma. Testes bioquímicos, incluindo um painel lipídico detalhado, creatinina sérica, albumina sérica, níveis de PCR de alta sensibilidade (PCR-us) e TFGe foram medidos. Além disso, os parâmetros inflamatórios baseados no hemograma completo, incluindo contagens de plaquetas, linfócitos e neutrófilos, foram calculados a partir de exames de hemograma completo de rotina. O INP foi calculado usando a seguinte equação: 10 × nível de albumina sérica (g/dl) + 0,005 × contagem total de linfócitos (por mm^2^). Exames de ecocardiografia transtorácica bidimensional foram realizados antes da AC e a FEVE foi determinada pelo método de Simpson modificado em cada paciente.

### Angiografia coronária e classificação de colaterais coronárias

Dependendo da preferência do operador, a técnica de Judkins foi utilizada para realizar a AC basal por via radial ou transfemoral. Para determinar a presença e o grau de CCC, as imagens de AC dos pacientes foram rigorosamente examinadas por dois cardiologistas intervencionistas seniores. A classificação Rentrop foi utilizada para graduar a CCC da seguinte forma: Grau 0= sem colateral visível na extremidade distal da obstrução, Grau 1= preenchimento de colateral por meio de vasos colaterais sem visualização do segmento epicárdico do ramo lateral, Grau 2= epicárdico parcial preenchimento de colaterais, com menor densidade e enchimento lento em relação ao vaso doador, e Grau 3= enchimento completo da artéria coronária epicárdica na extremidade distal da oclusão.^15^ Quando o paciente apresentava mais de um vaso OCT, o vaso com o mais alto grau de CCC foi selecionado. Com base nos dados angiográficos, os pacientes foram divididos em dois grupos; o grupo CCC ruim consistiu naqueles com colaterais de Grau 0 e I e o grupo CCC bom com colaterais de Grau II e III.

### Análise estatística

As análises estatísticas foram realizadas utilizando o software IBM SPSS versão 21.0 (Armonk, Nova York, EUA). A distribuição dos dados foi avaliada pelo teste de Kolmogorov-Smirnov. Os dados de medição com distribuição normal foram expressos como média ± desvio padrão, enquanto os com distribuição não normal foram expressos como mediana e intervalo interquartil (IQ) (25°-75°). Se os dados nos dois grupos tivessem distribuição normal, um teste t de amostra independente foi usado para análises diferentes. Caso os dados não se ajustassem à distribuição normal, foi utilizado o teste U de Mann-Whitney. As variáveis categóricas foram expressas como números e porcentagens e comparadas por meio do teste χ^2^. A comparação de médias entre múltiplos grupos foi realizada usando ANOVA unidirecional, seguida pelos testes post hoc de Bonferroni para análise de subgrupos. Para obter o valor de corte ideal e a área sob a curva (AUC) do INP para prever o grau de CCC, foi utilizada uma análise da curva ROC (característica de operação do receptor). Análises logísticas univariadas e multivariadas foram realizadas para explorar os potenciais fatores de risco para CCC deficiente em pacientes com SCE, e uma odds ratio (OR) com intervalo de confiança (IC) de 95% foi calculada. Variáveis com p < 0,10 na análise univariada foram inseridos em análise de regressão logística posterior. Um valor de p <0,05 foi considerado estatisticamente significativo.

## Resultados

**Figura 1:**
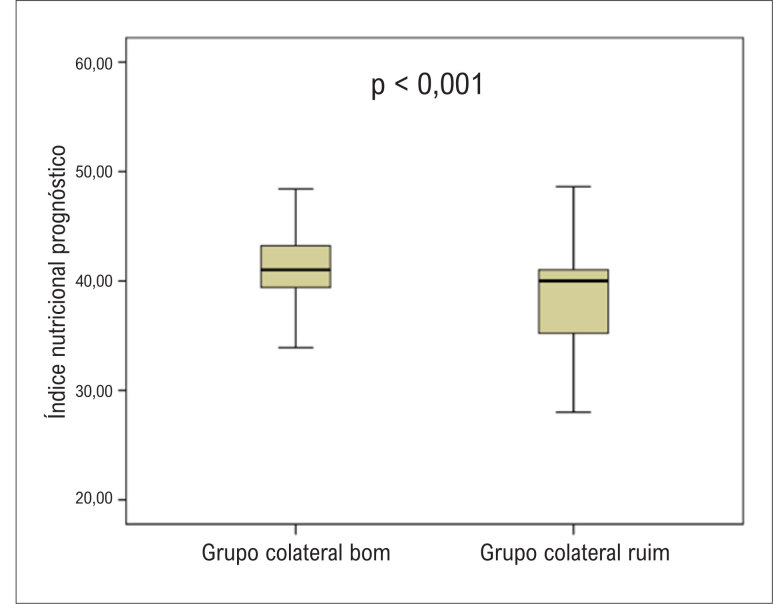
Comparação dos níveis do Curva ROC (Receiver Operating Characteristics) para (INP) entre pacientes com circulação colateral coronária bem e mal desenvolvida.

**Figura 2:**
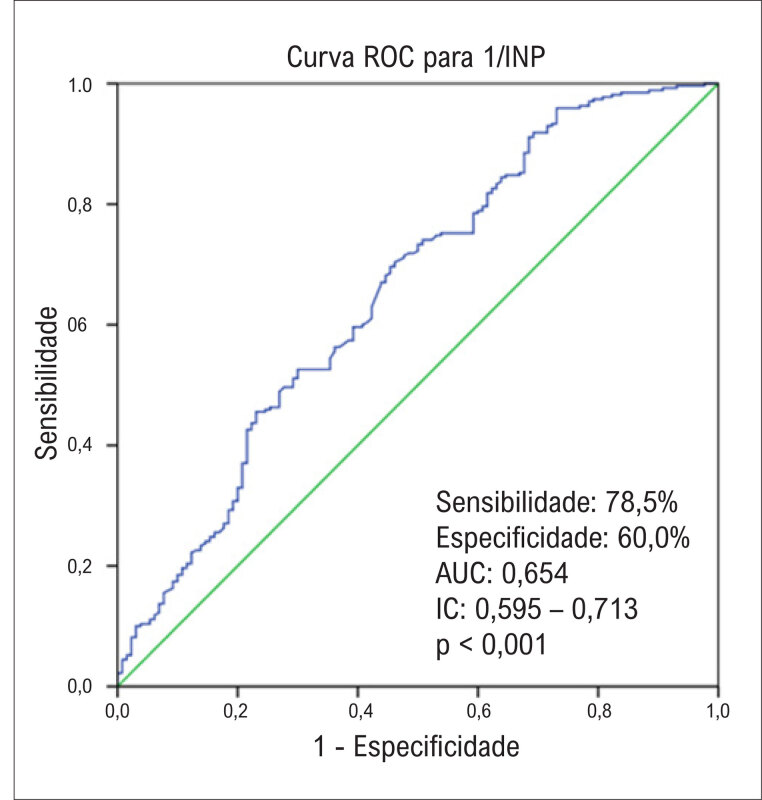
Curva ROC (Receiver Operating Characteristics) para determinação do melhor ponto de corte para índice nutricional prognóstico (INP) na predição da circulação colateral coronariana em pacientes com síndrome coronariana estável.

A média de idade da população estudada foi de 63±10 anos; 273 (68,3%) eram do sexo masculino. Os dados demográficos e clínicos basais da coorte do estudo são apresentados na Tabela 1 . Os pacientes que desenvolveram CCC deficiente foram indicados como sendo mais velhos, do sexo feminino e exibindo menos comorbidades (hipertensão arterial, tabagismo ativo, e hiperlipidemia). Os dados laboratoriais da população estudada são apresentados na Tabela 2 . Os pacientes que desenvolveram CCC deficiente apresentaram níveis séricos mais baixos de INP ( Figura 1 ), albumina sérica, colesterol total, colesterol de lipoproteína de baixa densidade, e maiores níveis de PCRus em comparação com aqueles que desenvolveram bom CCC. No entanto, os parâmetros do hemograma, incluindo contagens de linfócitos, glicose sérica, creatinina, colesterol de lipoproteína de alta densidade, triglicerídeos e TFGe foram comparáveis entre os dois grupos. Em análise adicional One-Way-ANOVA, os níveis de INP aumentaram gradualmente de Rentrop 0 a III (36,49±5,25 para Rentrop 0, 37,87±5,84 para Rentrop 1, 41,48±3,88 para Rentrop 3 e 42,17±4,59 para Rentrop 3). A análise post hoc com um ajuste de Bonferroni revelou que os níveis de INP aumentaram estatisticamente significativamente de Rentrop 0 para Rentrop 3 (p<0,001 para todos). Parâmetros significativos na análise univariada (idade, INP, sexo feminino, hipertensão arterial, colesterol total, tabagismo ativo, colesterol de lipoproteína de baixa densidade, colesterol de lipoproteína de alta densidade e hiperlipidemia) foram posteriormente inseridos na análise de regressão logística. Após análise multivariada, o INP (OR: 0,870) e o sexo feminino (OR: 1,845) foram encontrados como preditores independentes de CCC com baixo desenvolvimento ( Tabela 3 ). Conforme mostrado na figura 2 , na análise da curva ROC, o ponto de corte ideal para o INP prever CCC ruim foi 38,2. A AUC foi de 0,654, com boa sensibilidade e especificidade.

**Tabela 1:** Características demográficas e clínicas basais da população estudada

Características		Bom grupo colateral (n=270)	Grupo de garantias ruins (n=130)	Valor-p
Idade		62,28±9,87	64,68±11,46	0,032
Gênero feminino		72 (26,7%)	55 (42,3%)	0,002
História de hipertensão arterial (n, %)		170 (63%)	70 (53,8%)	0,081
História de diabetes mellitus (n, %)		84 (31,1%)	43 (33,1%)	0,692
Tabagismo ativo (n, %)		110 (40,7%)	36 (27,7%)	0,011
História de hiperlipidemia (n, %)		142 (52,6%)	54 (41,5%)	0,038
História familiar de doença coronariana (n, %)		73 (27%)	34 (26,2%)	0,852
Doença arterial coronariana multiarterial		130 (48,1%)	54 (41,5%)	0,214
**Vaso de oclusão total crônica**				
	Artéria descendente anterior esquerdo	64 (23,7%)	43 (33,1%)	
	Artéria circunflexa	45 (16,7%)	30 (23,1%)	0,012
	Artéria coronária direita	161 (59,6%)	57 (43,8%)	
Terapia prévia com betabloqueadores (n, %)		148 (54,8%)	71 (54,6%)	0,970
Terapia prévia com inibidores da enzima conversora de angiotensina (n, %)		166 (61,5%)	69 (53,1%)	0,110
Terapia prévia com clopidogrel (n, %)		92 (34,1%)	31 (23,8%)	0,138
Terapia prévia com estatinas (n, %)		166 (61,5%)	75 (57,7%)	0,468
Pressão arterial sistólica (mmHg)		126,7±15,9	125,9±15,6	0,610
Pressão arterial diastólica (mmHg)		87,13±11,56	78,52±10,25	0,231
Fração de ejeção do ventrículo esquerdo (%)		47,72±11,34	46,43±12,37	0,302
**Número de vasos com oclusão total crônica**				
	1	167 (61,9%)	86 (66,2%)	
	2	53 (19,6%)	30 (23,1%)	0,132
	3	50 (18,5%)	14 (10,8%)	

**Table 2:** Dados laboratoriais dos pacientes do estudo

Variáveis	Grupo Colateral Bom (n=270)	Grupo Colateral Ruim (n=130)	Valor p
Índice nutricional prognóstico	41,23±3,85	38,29±5,58	<0,001
Creatinina sérica (mg/dl)	0,97±0,27	0,99±0,25	0,692
Taxa de filtração glomerular (mL/min/1,73 m^2^)	86,09±16,43	82,54±17,9	0,158
Contagem de linfócitos (×109/L)	2,71±0,89	2,55±0,94	0,112
Contagem de glóbulos brancos (×109/L)	8,07±2,06	8±2,35	0,747
Contagem de plaquetas (×109/L)	254,06±83,7	250,55±65,09	0,646
Hemoglobina (g/dL)	14,69±8,34	13,58±2	0,133
Glicose sérica (mg/dl)	124,81±63,96	132,28±80,24	0,354
Colesterol total (mg/dl)	191,45±55,72	178,98±47,19	0,028
Colesterol de lipoproteína de baixa densidade (mg/dl)	115,56±39,09	104,49±38,44	0,008
Colesterol lipoproteico de alta densidade (mg/dl)	41,16±22,63	43,52±27,24	0,392
Triglicerídeos (mg/dl)	154 (110-219)	144 (101-190)	0,186
Proteína C reativa de alta sensibilidade (mg/dl)	3,67 (2,12-6,98)	4,69 (2,32-12,8)	0,028
Albumina (g/dL)	4,12±0,38	3,87±0,54	<0,001

**Tabela 3:** Preditores independentes de colaterais ruins na análise multivariada

Variável	Odds ratio	Intervalo de confiança de 95%	Valor p
Índice nutricional prognóstico	0,870	(0,822-0,922)	<0,001
Idade	0,994	(0,970-1,018)	0,617
Gênero feminino	1,845	(1,056-3,223)	0,031
Hipertensão	1,574	(0,931-2,658)	0,098
Fumante ativo	1,398	(0,786-2,487)	0,255
Colesterol total	1,002	(0,994-1,010)	0,694
Colesterol de lipoproteína de baixa densidade	0,991	(0,979-1,002)	0,098
Proteína C reativa de alta sensibilidade	1,019	(1,000-1,038)	0,051
Hiperlipidemia	1,154	(0,702-1,897)	0,572

## Discussão

Até onde sabemos, este é o primeiro estudo a explorar o INP como um preditor independente de formação de colaterais coronárias. Demonstramos que os níveis de INP estavam significativamente associados ao grau de colateralização avaliado pelo escore Rentrop. Valores mais baixos de INP foram independentemente associados ao CCC pouco desenvolvido em pacientes com SCE com OTC.

O desenvolvimento da CCC é uma resposta adaptativa à isquemia miocárdica aguda ou crônica e serve como um canal que liga a artéria coronária epicárdica significativamente estreitada.^16^ Colaterais bem desenvolvidas podem proteger o miocárdio da isquemia, melhorar a contratilidade miocárdica residual e, assim, reduzir sintomas anginosos.^17^ Além disso, muitos estudos demonstraram que boas colaterais melhoram o prognóstico em pacientes com SCE.^18^ No entanto, sabemos que o grau de formação de CCC varia entre os pacientes, apesar do mesmo grau de estreitamento ou oclusão nas artérias coronárias. Nesse contexto, vários fatores como idade, diabetes, hipercolesterolemia, hipertensão, duração e/ou grau de oclusão coronariana, funções endoteliais e estresse oxidativo podem afetar a formação do CCC.^19 – 21^ Além disso, muitos marcadores inflamatórios têm sido sugeridos em associação com o desenvolvimento de CCC.^3 , 4^ Recentemente, o INP tem sido recomendado para representar o estado inflamatório, e um INP mais baixo está significativamente associado a numerosos eventos cardiovasculares adversos.^9 – 14^ No entanto, nenhum estudo foi feito sobre a associação entre INP níveis e CCC em pacientes com SCE com OCT até o momento. Aqui, o presente estudo mostrou que níveis mais baixos de INP estão independentemente associados a CCC deficiente em pacientes com SCE. Assim, o cálculo do INP pode ser um valioso biomarcador do grau de CCC nesses pacientes. Sugerimos que esta associação entre INP e CCC pode envolver alguns mecanismos.

Os hepatócitos sintetizam albumina e desempenham um papel crucial nas vias inflamatórias agudas e crônicas.^22^ A albumina sérica também possui muitas propriedades fisiológicas, incluindo atividade antioxidante, antiinflamatória, anticoagulante e antiagregante.^23 , 24^ A concentração de albumina sérica está inversamente associada à extensão e carga de aterosclerose e prognóstico em pacientes com DAC.^25^ Níveis mais baixos de albumina sérica também podem provocar aumento da viscosidade sanguínea, o que torna a lipoproteína-colesterol de baixa densidade sensível à modificação oxidativa, provocando dano endotelial vascular.^26^ Além disso, níveis baixos de albumina podem causar disfunção endotelial reduzindo a produção de óxido nítrico, que é necessário para a angiogênese, remodelação vascular e desenvolvimento de CCC.^22^ À luz desses dados, podemos especular que o mau desenvolvimento da CCC em pacientes com baixa albumina sérica pode estar relacionado à disfunção endotelial e à diminuição da produção de óxido nítrico. Por outro lado, as células linfocitárias têm propriedades anti-inflamatórias e contagens baixas de linfócitos em muitas doenças cardiovasculares têm sido independentemente associadas a um mau prognóstico.^27 – 29^ As células linfocitárias têm um papel fundamental no início e manutenção de respostas neovasculares, e os sinais inflamatórios recrutam linfócitos para áreas de neovascularização, que atua como fonte de fatores angiogênicos.^30^ Considerando esses dados, sugerimos que baixas contagens de linfócitos podem afetar adversamente o desenvolvimento da CCC.

Tomados em conjunto, valores mais baixos de INP devido à diminuição dos níveis de albumina e da contagem de linfócitos estão relacionados ao aumento da carga inflamatória e à má colateralização coronariana.

### Limitações do estudo

Existem algumas limitações no estudo atual. Primeiro, este é um estudo retrospectivo unicêntrico com um tamanho de amostra relativamente pequeno; portanto, nosso estudo não pode elucidar os mecanismos precisos que ligam o menor INP e o fraco desenvolvimento do CCC na SCE. Em segundo lugar, a classificação do CCC baseou-se exclusivamente na classificação Rentrop, o que significa que os pequenos vasos microscópicos podem não ser visíveis angiograficamente. Conforme nosso protocolo de estudo, avaliamos colaterais apenas com a classificação Rentrop. Seria ótimo se pudéssemos avaliar também as garantias com outro sistema de classificação, por exemplo, o sistema Werner. Terceiro, o presente estudo não provou causalidade, mas sim detectou associações. Esses dados não provam que o baixo INP tenha causado colaterais coronárias pouco desenvolvidas. Finalmente, a medição do INP foi realizada apenas uma vez, mas o desenvolvimento do CCC é um processo que continuará por muitos anos. No entanto, este estudo ainda é valioso para destacar a importância potencial do INP na avaliação da CCC em pacientes com SCE.

## Conclusão

Os níveis de INP foram associados ao desenvolvimento de CCC, e níveis mais baixos de INP foram independentemente correlacionados com CCC deficiente. Isto sugere que o INP poderia ser usado como um novo indicador para avaliar as colateralizações em pacientes com SCE.
