# Supplementary material for: O Índice Nutricional Prognóstico está Associado ao Grau de Circulação Colateral Coronariana em Pacientes com Angina Estável e Oclusão Total Crônica
Source: Arq Bras Cardiol. 2024 Mar 21;121(2):e20230765. [Article in Portuguese] doi: 10.36660/abc.20230765 (PMC12092056; doi:10.36660/abc.20230765)
Supplement: Translation

# Translation: Prognostic Nutritional Index is Associated with the Degree of Coronary Collateral Circulation in Stable Angina Patients with Chronic Total Occlusion

**Authors:** Kerim Esenboga, Alparslan Kurtul, Yakup Yunus Yamanturk, Volkan Kozluca, Eralp Tutar

**Affiliations:** 1 Ankara Turkey Ankara University Faculty of Medicine – Cardiology, Ankara – Turkey; 2 Hatay Turkey Mustafa Kemal University Tayfur Ata Sokmen Faculty of Medicine – Department of Cardiology, Hatay – Turkey

**Keywords:** Collateral Circulation, Nutrition Assessment, Inflammation

## Abstract

**Background:**

Coronary collateral circulation (CCC) can effectively improve myocardial blood supply to the area of CTO (chronic total coronary occlusion) and can, thus, improve the prognosis of patients with stable coronary syndrome (SCS). The degree of inflammation and some inflammation markers were associated with the development of collaterals.

**Objective:**

To investigate whether prognostic nutritional index (PNI) has an association with the development of CCC in patients with SCS.

**Methods:**

A total of 400 SCS patients with the presence of CTO in at least one major epicardial coronary artery were included in this study. The patients were divided into two groups according to the Rentrop score. Scores of 0 to 1 were considered poor developed CCC, and scores of 2 to 3 were accepted as good developed CCC. Statistical significance was set as a p-value < 0.05 for all analyses.

**Results:**

The mean age of the study cohort was 63±10 years; 273 (68.3%) were males. The poor-developed CCC group had a significantly lower PNI level compared with the good-developed CCC group (38.29±5.58 vs 41.23±3.85, p< 0.001). In the multivariate analysis, the PNI (odds ratio 0.870; 95% confidence interval 0.822-0.922; p< 0.001) was an independent predictor of poorly developed CCC.

**Conclusion:**

The PNI can be used as one of the independent predictors of CCC formation. It was positively associated with the development of coronary collaterals in SCS patients with CTO.

**Figure:** Summary of study design and results.

## Introduction

Coronary collateral circulation (CCC) is usually an adaptive mechanism during chronic myocardial ischemia to supply blood flow in the ischemic territory.^1^The development of CCC can maintain coronary blood flow to some extent, alleviate anginal complaints, provide myocardial preservation in the face of acute ischemia, preserve myocardial function, and also improve survival in stable coronary syndrome (SCS) patients.^2^Although the exact mechanisms of coronary collateralization development in patients with SCS are still conflicting and inconclusive, several studies suggest that systemic inflammatory markers such as the neutrophil/lymphocyte ratio (NLR) and C-reactive protein (CRP) are associated with the development of collaterals.^3 , 4^

Previous studies have reported that immunological and nutritional statuses are closely associated with cardiovascular disease development, progression, and prognosis.^5 , 6^Recently, investigation on the prognostic nutritional index (PNI) has become very popular. The PNI, which is calculated from the serum albumin level and total lymphocyte cell count in the peripheral blood, is an index that indicates chronic inflammation, immune system and nutritional status and has a prognostic value in various cancers.^7 , 8^Recently, many studies have reported that the PNI is closely related to cardiovascular disease, and a lower PNI is significantly associated with increased adverse clinical outcomes, including mortality in patients with SCS, heart failure, aortic dissection, and acute myocardial infarction.^9 – 14^However, the association between PNI and CCC has not been investigated yet.

The goal of the current study is, therefore, to explore whether there is a correlation between PNI and the degree of CCC development in patients with SCS.

## Methods

### Study patients

The study population consisted of 442 SCS patients who underwent coronary angiography (CA) and were detected with chronic total coronary occlusion (CTO) in any coronary artery from May 2017 to May 2021. The CA was performed to investigate occlusive coronary artery disease based on clinical signs such as the presence of typical anginal symptoms, abnormal or suspicious treadmill tests, and abnormal myocardial perfusion scintigraphy findings suggestive of myocardial ischemia. Patients with prior myocardial infarctions, prior coronary artery bypass graft surgery, history of percutaneous coronary intervention, significant heart failure (left ventricular ejection fraction [LVEF] ≤35%), severe chronic kidney disease (estimated glomerular filtration rate [eGFR] <30 mL/min/1.73 m²), severe valvular dysfunction, severe chronic liver disease, hematological disorders, active an immunologic/infectious/inflammatory disease, and patients with malignancy were not included in the study. Forty-two patients were excluded due to exclusion criteria (10 patients with previous history of myocardial infarction or percutaneous coronary intervention; 10 patients with severe chronic kidney disease; 5 patients with heart failure; 5 patients with valvular heart disease; 3 patients with inflammatory diseases; 2 patients with malignancy; 5 patients with chronic liver disease; and 2 patient with hematological disorders). The remaining 400 patients were included in the final analysis. In total, 130 patients were allocated to the poor CCC group, while 270 patients were allocated to the good CCC group.

Baseline clinical data and atherosclerotic cardiovascular disease risk factors were noted for all patients. Arterial hypertension was considered in patients with repeated blood pressure measurements >140/90 mmHg or those who were already using antihypertensive pills. Diabetes mellitus was described as having a fasting serum glucose level >126 mg/dL and postprandial glucose >200 mg/dL on repeated measurements or current use of antidiabetic therapy. An increased total cholesterol level of more than 200 mg/dL and/or the usage of anti-hyperlipidemic medications were used to characterize hypercholesterolemia. The definition of a family history of coronary artery disease (CAD) was a history of CAD or sudden cardiac death in first-degree relatives who were under the age of 55 for men and 65 for women.

Antecubital venous blood samples were taken after at least 12 hours of fasting before the CA. Beckman colter analyzer was used to measure the routine hemogram parameters. Biochemical tests, including a detailed lipid panel, serum creatinine, serum albumin, high sensitivity CRP (hs-CRP) levels, and eGFR were measured. Also, the complete blood count-based inflammatory parameters, including platelet, lymphocyte, and neutrophil counts, were calculated from routine complete blood count tests. The PNI was calculated using the following equation: 10 × serum albumin level (g/dl) + 0.005 × total lymphocyte count (per mm^2^). Two-dimensional transthoracic echocardiography tests were done before the CA, and the LVEF was determined by using the modified Simpson method in each patient.

### Coronary angiography and grading of coronary collaterals

Depending on the operator's preference, the Judkins technique was used to perform the baseline CA either through the transradial or transfemoral approach. To determine the presence and degree of CCC, the CA images of the patients were rigorously examined by two senior interventional cardiologists. The Rentrop classification was used to grade the CCC as follows: Grade 0= no visible collateral at the distal end of the obstruction, Grade 1= filling in collateral by means of collateral vessels without visualizing the side branch epicardial segment, Grade 2= partial epicardial filling of collaterals, with lower density and slow filling compared with the donor vessel, and Grade 3= complete filling of the epicardial coronary artery at the distal end of the occlusion.^15^When the patient had more than one CTO vessel, the vessel with the highest degree of CCC was selected. Based on angiographic data, patients were divided into two groups; poor CCC group consisted of those with Grade 0 and I collateral and good CCC group with Grade II and III collaterals.

### Statistical analysis

Statistical analyses were performed using the IBM SPSS version 21.0 software (Armonk, New York, USA). The distribution of the data was assessed using the Kolmogorov-Smirnov test. Measurement data with normal distribution were expressed as mean ± standard deviation, while the ones with non-normal distribution were expressed as median and interquartile range (IQR) (25th–75th). If data in the two groups was the normal distribution, an independent sample t-test is used for different analyses. If data do not fit the normal distribution, the Mann-Whitney U test was used. Categorical variables were expressed as numbers and percentages and compared using aχ2-test. Comparison of means among multiple groups was conducted by using one-way ANOVA, followed by Bonferroni's posthoc tests for subgroup analysis. To obtain the optimal cut-off value and the area under the curve (AUC) of PNI for predicting the degree of CCC, a receiver operating characteristic (ROC) curve analysis was used. Univariate and multivariate logistic analyses were performed to explore the potential risk factors for poor CCC in SCS patients, and an odds ratio (OR) with a 95% confidence interval (CI) was calculated. Variables with a p < 0.10 in the univariate analysis were entered into further logistic regression analysis. A p-value <0.05 was considered statistically significant.

## Results

The mean age of the study population was 63±10 years; 273 (68.3%) were males. The baseline demographic and clinical data of the study cohort are presented in Table 1 . Patients who developed poor CCC were indicated to be older and female gender, and they exhibited fewer comorbidities (arterial hypertension, active smoking, and hyperlipidemia). Laboratory data of the study population are presented in Table 2 . Patients who developed poor CCC had lower serum levels of PNI ( Figure 1 ), serum albumin, total cholesterol, low-density lipoprotein cholesterol, and higher hs-CRP levels compared with those who developed good CCC. However, CBC parameters, including lymphocyte counts, serum glucose, creatinine, high-density lipoprotein cholesterol, triglycerides, and eGFR were comparable between the two groups. In additional One-Way-ANOVA analysis, the PNI levels gradually increased from Rentrop 0 to III (36.49±5.25 for Rentrop 0, 37.87±5.84 for Rentrop 1, 41.48±3.88 for Rentrop 3, and 42.17±4.59 for Rentrop 3). Post hoc analysis with a Bonferroni adjustment revealed that PNI levels were statistically significantly increased from Rentrop 0 to Rentrop 3 (p< 0.001 for all). Significant parameters in the univariate analysis (age, PNI, female gender, arterial hypertension, total cholesterol, active smoking, low-density lipoprotein cholesterol, high-density lipoprotein cholesterol, and hyperlipidemia) were further entered into the logistic regression analysis. After multivariate analysis, PNI (OR: 0.870) and female gender (OR: 1.845) were found as independent predictors of poor developed CCC ( Table 3 ). As shown in Figure 2 , In ROC curve analysis, the optimal cut-off for PNI to predict poor CCC was 38.2. The AUC was 0.654, with good sensitivity and specificity.

**Table 1:** Baseline demographic and clinical characteristics of the study population

Characteristics		Good collateral group (n=270)	Poor collateral group (n=130)	p value
Age		62.28±9.87	64.68±11.46	0.032
Female gender		72 (26.7%)	55 (42.3%)	0.002
History of arterial hypertension (n, %)		170 (63%)	70 (53.8%)	0.081
History of diabetes mellitus (n, %)		84 (31.1%)	43 (33.1%)	0.692
Active smoking (n, %)		110 (40.7%)	36 (27.7%)	0.011
History of hyperlipidemia (n, %)		142 (52.6%)	54 (41.5%)	0.038
Family history of coronary heart disease (n, %)		73 (27%)	34 (26.2%)	0.852
Multivessel coronary artery disease		130 (48.1%)	54 (41.5%)	0.214
**Chronic total occlusion vessel**				
	Left anterior descending	64 (23.7%)	43 (33.1%)	
	Circumflex artery	45 (16.7%)	30 (23.1%)	0.012
	Right coronary artery	161 (59.6%)	57 (43.8%)	
Prior beta-blocker therapy (n, %)		148 (54.8%)	71 (54.6%)	0.970
Prior angiotensin-converting enzyme inhibitors therapy (n, %)		166 (61.5%)	69 (53.1%)	0.110
Prior clopidogrel therapy (n, %)		92 (34.1%)	31 (23.8%)	0.138
Prior statin therapy (n, %)		166 (61.5%)	75 (57.7%)	0.468
Systolic blood pressure (mmHg)		126.7±15.9	125.9±15.6	0.610
Diastolic blood pressure (mmHg)		87.13±11.56	78.52±10.25	0.231
Left ventricular ejection fraction (%)		47.72±11.34	46.43±12.37	0.302
**Number of chronic total occlusion vessel**				
	1	167 (61.9%)	86 (66.2%)	
	2	53 (19.6%)	30 (23.1%)	0.132
	3	50 (18.5%)	14 (10.8%)	

**Table 2:** Laboratory data of the study patients

Variables	Good collateral group (n=270)	Poor collateral group (n=130)	p value
Prognostic nutrional index	41.23±3.85	38.29±5.58	<0.001
Serum creatinine (mg/dl)	0.97±0.27	0.99±0.25	0.692
Glomerular filtration rate (mL/min/1.73 m^2^)	86.09±16.43	82.54±17.9	0.158
Lymphocyte count (×10^9^/L)	2.71±0.89	2.55±0.94	0.112
White blood cell count (×10^9^/L)	8.07±2.06	8±2.35	0.747
Platelet count (×10^9^/L)	254.06±83.7	250.55±65.09	0.646
Hemoglobin (g/dL)	14.69±8.34	13.58±2	0.133
Serum glucose (mg/dl)	124.81±63.96	132.28±80.24	0.354
Total cholesterol (mg/dl)	191.45±55.72	178.98±47.19	0.028
Low-density lipoprotein cholesterol (mg/dl)	115.56±39.09	104.49±38.44	0.008
High-density lipoprotein cholesterol (mg/dl)	41.16±22.63	43.52±27.24	0.392
Triglyceride (mg/dl)	154 (110-219)	144 (101-190)	0.186
High-sensitivity C-reactive protein (mg/dl)	3.67 (2.12-6.98)	4.69 (2.32-12.8)	0.028
Albumin (g/dL)	4.12±0.38	3.87±0.54	<0.001

**Table 3:** Independent predictors of poor collaterals in multivariate analysis

Variable	Odds ratio	95% Confidence interval	p value
Prognostic nutrional index	0.870	(0.822-0.922)	<0.001
Age	0.994	(0.970-1.018)	0.617
Female gender	1.845	(1.056-3.223)	0.031
Hypertension	1.574	(0.931-2.658)	0.098
Active smoking	1.398	(0.786-2.487)	0.255
Total cholesterol	1.002	(0.994-1.010)	0.694
Low-density lipoprotein cholesterol	0.991	(0.979-1.002)	0.098
High-sensitivity C-reactive protein	1.019	(1.000-1.038)	0.051
Hyperlipidemia	1.154	(0.702-1.897)	0.572

**Figure 1:** Comparison of the prognostic nutritional index (PNI) levels between patients with good and poor developed coronary collateral circulation.

**Figure 2:** Receiver operating characteristics (ROC) curve for determination of the best cut-off for prognostic nutritional index (PNI) in predicting coronary collateral circulation in patients with stable coronary syndrome.

## Discussion

To the best of our knowledge, this is the first study to explore PNI as an independent predictor of coronary collateral formation. We demonstrated that PNI levels were significantly associated with the degree of collateralization evaluated by the Rentrop score. Lower PNI values were independently associated with poorly developed CCC in SCS patients with CTO.

CCC development is an adaptive response to acute or chronic myocardial ischemia and serves as a conduit bridging the significantly narrowed epicardial coronary artery.^16^Well-developed collaterals may protect the myocardium from ischemia, improve residual myocardial contractility, and thus reduce anginal symptoms.^17^Moreover, many studies have shown that good collaterals improve prognosis in patients with CCS.^18^However, we know that the degree of CCC formation varies between patients despite the same degree of narrowing or occlusion in the coronary arteries. In this context, several factors such as age, diabetes, hypercholesterolemia, hypertension, duration and/or degree of coronary occlusion, endothelial functions, and oxidative stress may affect CCC formation.^19 – 21^In addition, many inflammatory markers have been suggested in association with the development of CCC.^3 , 4^Recently, the PNI has been recommended to represent the inflammatory status, and a lower PNI is significantly associated with numerous adverse cardiovascular events.^9 – 14^However, no study has been done on the association between PNI levels and CCC in CCS patients with CTO so far. Herein, the present study has shown that lower PNI levels are independently associated with poor CCC in SCS patients. Thus, calculating PNI may be a valuable biomarker of the degree of CCC in these patients. We suggest that this association between PNI and CCC may involve some mechanisms.

Hepatocytes synthesize albumin and play a crucial role in acute and chronic inflammatory pathways.^22^Serum albumin also has many physiological properties, including antioxidant, antiinflammatory, anticoagulant, and antiaggregant activity.^23 , 24^Serum albumin concentration is inversely associated with the extent and burden of atherosclerosis and prognosis in patients with CAD.^25^Lower serum albumin levels can also bring about increased blood viscosity, which makes low-density lipoprotein-cholesterol sensitive to the oxidative modification, provoking vascular endothelial damage.^26^Additionally, low albumin levels may cause endothelial dysfunction by reducing the production of nitric oxide, which is necessary for angiogenesis, vascular remodeling, and CCC development.^22^In light of these data, we can speculate that poor CCC development in patients with low serum albumin may be related to endothelial dysfunction and decreased nitric oxide production. On the other hand, lymphocyte cells have antiinflammatory properties and low lymphocyte counts in many cardiovascular diseases have been independently linked with poor prognosis.^27 – 29^Lymphocyte cells have a pivotal role in initiating and maintaining neovascular responses, and inflammatory signals recruit lymphocytes into areas of neovascularization, which act as a source of angiogenic factors.^30^Considering these data, we suggest that low lymphocyte counts may adversely affect the development of CCC.

Taken together, lower PNI values because of decreased albumin levels and lymphocyte counts are related to increased inflammatory burden and poor coronary collateralization.

### Study limitations

There are some limitations in the current study. First, this is a single-center retrospective study with a relatively small sample size; therefore, our study cannot elucidate the precise mechanisms linking lower PNI and poor CCC development in SCS. Second, the CCC grading was based solely on the Rentrop classification, which means that the small, microscopic vessels may not be visible angiographically. As per our study protocol, we evaluated collaterals only with the Rentrop classification. It would be great if we could also evaluate collaterals with another classification system, for example, the Werner system. Third, the present study did not prove causality but rather detected associations. These data do not prove that low PNI caused poor-developed coronary collaterals. Finally, PNI measurement was performed only once, but CCC development is a process that will continue for many years. However, this study is still valuable in highlighting the potential importance of PNI in assessing CCC in SCS patients.

## Conclusion

PNI levels were associated with CCC development, and lower PNI levels were independently correlated to poor CCC. This suggests that PNI could be used as a new indicator to assess the collateralizations in patients with SCS.
